# User Experiences With a Type 2 Diabetes Coaching App: Qualitative Study

**DOI:** 10.2196/16692

**Published:** 2020-07-17

**Authors:** Shaira Baptista, Greg Wadley, Dominique Bird, Brian Oldenburg, Jane Speight, The My Diabetes Coach Research Group

**Affiliations:** 1 Melbourne School of Population and Global Health Melbourne Australia; 2 Australian Centre for Behavioural Research in Diabetes Melbourne Australia; 3 School of Computing and Information Systems The University of Melbourne Melbourne Australia; 4 Centre for Health Services Research The University of Queensland Brisbane Australia; 5 See Authors' Contributions section

**Keywords:** type 2 diabetes, mobile phone, mobile apps, mHealth, smartphone, self-management

## Abstract

**Background:**

Diabetes self-management apps have the potential to improve self-management in people with type 2 diabetes (T2D). Although efficacy trials provide evidence of health benefits, premature disengagement from apps is common. Therefore, it is important to understand the factors that influence engagement in real-world settings.

**Objective:**

This study aims to explore users’ real-world experiences with the *My Diabetes Coach* (MDC) self-management app.

**Methods:**

We conducted telephone-based interviews with participants who had accessed the MDC self-management app via their smartphone for up to 12 months. Interviews focused on user characteristics; the context within which the app was used; barriers and facilitators of app use; and the design, content, and delivery of support within the app.

**Results:**

A total of 19 adults with T2D (8/19, 42% women; mean age 60, SD 14 years) were interviewed. Of the 19 interviewees, 8 (42%) had T2D for <5 years, 42% (n=8) had T2D for 5-10 years, and 16% (n=3) had T2D for >10 years. In total, 2 themes were constructed from interview data: (1) the moderating effect of diabetes self-management styles on needs, preferences, and expectations and (2) factors influencing users’ engagement with the app: one size does not fit all.

**Conclusions:**

User characteristics, the context of use, and features of the app interact and influence engagement. Promoting engagement is vital if diabetes self-management apps are to become a useful complement to clinical care in supporting optimal self-management.

**Trial Registration:**

Australia New Zealand Clinical Trials Registry CTRN126140012296; URL https://www.anzctr.org.au/Trial/Registration/TrialReview.aspx?id=366925&isReview=true

## Introduction

### Background

By 2045, 693 million people will be living with diabetes, the majority with type 2 diabetes (T2D) [1]. Diabetes self-management behaviors, including blood glucose monitoring, healthy eating, being physically active and taking prescribed medications, can improve diabetes-related outcomes, reduce complications, and improve quality of life, but these behaviors can be difficult to initiate and sustain [2]. Diabetes self-management education and ongoing support are critical for establishing and maintaining self-care routines [3]. However, the uptake of face-to-face educational programs is low because of several factors, including difficulty in attending because of medical, financial, or transport issues; lack of perceived benefits; and shame and stigma [4-7]. Furthermore, the provision of ongoing support is difficult because of resource constraints and issues of reach and scalability [5]. An increasingly common strategy to address these challenges has been to use smartphone apps as a means to deliver diabetes education and self-management support to complement clinical care.

The evidence for the efficacy and acceptability of diabetes self-management apps is increasingly robust [8-11]. However, research trials typically focus on overall efficacy, not individual differences in user experiences, and cannot shed light on factors that influence engagement [12-14]. This is a gap that needs to be addressed if apps that demonstrate efficacy in controlled trial settings are to be translated into effective real-world interventions [15,16].

The lower engagement, or lack of thereof, with diabetes self-management apps is often attributed to a mismatch between what people with T2D want and the functions provided by apps, loss of motivation, and the difficulty integrating app use into everyday life [17-22]. Research suggests that multiple factors, including treatment, attitudes to self-management, and existing knowledge, influence the needs and preferences of people with T2D [22]. For example, people with newly diagnosed diabetes favor apps that educate them about diabetes, whereas those with more experience of living with and managing diabetes express frustration with basic education materials and are keen to see more *cutting edge* news and links for further reading [23-25]. Those who have been living with diabetes for longer engage with technology to refine care routines, whereas those less experienced use diabetes self-management tools to establish routines, for example by troubleshooting out-of-range blood glucose readings [20,26]. Finally, those with more experience are less willing to explore new options, including apps, especially if the benefits are uncertain, and the effort is substantial [27]. Unfortunately, participants in these studies were asked either to give feedback on apps they had not used before or to use unfamiliar devices. These limitations precluded an in-depth examination of user experiences over time and in the context of participants’ everyday lives.

### Objectives

Therefore, this study aimed to investigate users’ experiences of a diabetes self-management app (*My Diabetes Coach* [MDC]) accessed via personal devices and used in the context of everyday life over a prolonged period and to understand the interplay between users’ characteristics, needs, and preferences and engagement with a diabetes self-management app.

## Methods

### Design and Ethics

This qualitative study was a substudy of a randomized controlled trial testing the efficacy of a T2D self-management app MDC. The trial was conducted from 2014 to 2018 (Australia New Zealand Clinical Trials Registry ID ACTRN12614001229662) [28,29]. The University of Melbourne’s human research ethics committee approved this study (HREC number: 1442433). In-depth, semistructured interviews were conducted to evaluate the MDC app in terms of users’ experiences. We used a qualitative approach to explore subjective perspectives constructed from the experience of people with T2D using a self-management app in the context of their everyday lives [30]. This report is consistent with the consolidated criteria for reporting qualitative research checklist (Multimedia Appendix 1) [31].

### Intervention Description

**Figure 1:**
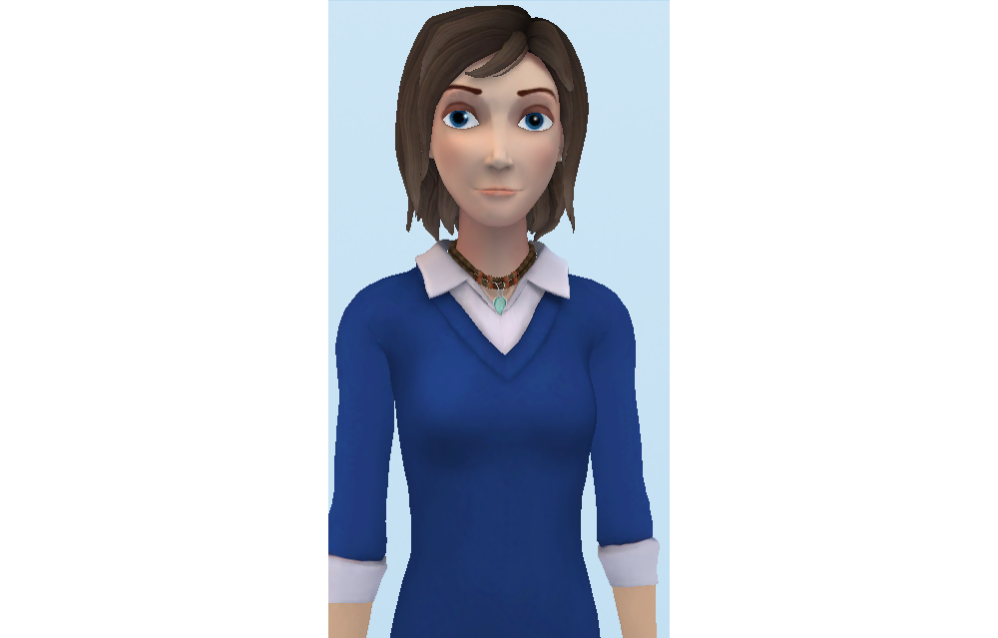
Embodied conversational agent Laura.

The MDC app was designed to provide education, support, and feedback on diabetes self-care using weekly sessions or *appointments* with an embodied conversational agent *Laura* (Figure 1). Laura had human-like characteristics and mimicked human conversation using interactive voice recognition (IVR) and a database of prerecorded conversational elements. Laura conversed with users either via spoken voice or text, using sophisticated script logic. The app’s script logic was personalized by incorporating information and targets provided by users’ health care professionals (eg, blood glucose monitoring targets). Users were able to respond to Laura by speaking, inputting text, or touching an option on the screen. The program was designed to enable responses made in a preceding session to dictate the direction of the next session with the user, enabling a high degree of personalization.

The first *appointment* with Laura was scheduled to suit the user and thereafter occurred at the same time every week, with some flexibility, enabling users to complete their appointment up to 48 hours after the planned time. Users could choose a particular module from those available but were required to complete the module over a series of sessions before moving to a new one. Available modules included blood glucose monitoring, nutrition, physical activity, medication taking, and foot care. The app applied several gamification elements, including goal setting, monitoring of progress, feedback, and quizzes [32].

Throughout the trial, users had access to a program coordinator to assist them with technical difficulties. They were also given an Accu-Chek Advantage blood glucose monitoring device with Bluetooth capabilities (Roche Diabetes Care), enabling the automated upload of glucose data to the MDC app. Finally, the app had inbuilt links to a website with diabetes resources and a user guide for the app.

### Study Participants and Recruitment

Invitations to participate in the MDC trial were sent by mail to adults with T2D (in New South Wales, Queensland, Victoria, and Western Australia) registered with the National Diabetes Services Scheme (NDSS). Participants were eligible if they were adults aged 18 years or older, diagnosed with T2D, registered with the NDSS for <10 years, had access to a smartphone (with an operating system of at least iOS 8.0 for Apple devices or OS 4.2 for Android), and fluent in the English language. The exclusion criteria were as follows: women who were pregnant or planning to become pregnant; individuals reporting severe comorbid conditions that would prevent participation in the trial; and individuals on nonstable doses of diabetes-related medications.

Interview participants for the qualitative study were recruited from the intervention arm of the MDC trial, all of whom had accessed the MDC app for up to 12 months. Purposive sampling was used to achieve variation in user characteristics, including age, gender, education, occupation location, duration of T2D, and use of the app (operationalized as the number of completed chats).

### Data Collection

Participants were sent a plain language statement describing the study and were required to provide written consent. Participant characteristics were collected at baseline via a self-report questionnaire, including demographic and clinical details and current health app use.

An interview guide was developed to include questions about the user’s self-reported diabetes expertise, how they managed their diabetes, when and how they engaged with the app, and their experiences using it. In-depth semistructured interviews were conducted through telephone (by SB) and recorded using SmartInteraction Suite, a cloud architecture voice recording solution (CTI Group). SB has several years of experience in diabetes-related research, including conducting telephone interviews. She worked as a research assistant on the MDC project and was involved with program development, participant recruitment, and data collection. Many of the participants had previously interacted with her. At the beginning of each interview, SB summarized the research and reasons for her interest in it.

The first 2 interviews were analyzed, and changes were made to the interview guide to capture additional information on the context of use and feedback on the timing and delivery of sessions. Data included researcher observations and postinterview notes. Data collection continued until saturation was achieved (19 interviews), as indicated by the recurrence of themes and no new themes emerging. Recordings were stored in a secure cloud-based location and transcribed verbatim by an accredited transcription service with privacy certification. During each interview, SB kept notes of points of interest and used these as prompts. Immediately after each interview, SB prepared a written summary of the interview and relevant observations. These were used to communicate interim findings to the wider research team. When appropriate, additional questions were added to the interview guide, allowing for further exploration of issues raised by participants that were relevant to the research aims. These notes were also used to guide meaningful interpretation of data during data analysis.

### Data Analysis

Descriptive statistics were computed for demographic and clinical characteristics and current app use using SPSS version 25 (IBM Corp). Data are presented as mean (SD) or number (percentage). Raw interview data were imported into NVivo 11 (QSR International) for coding and analysis. We followed 6 steps for the thematic analysis with the development of themes guided by a priori objectives identified in the aims: (1) data familiarization, (2) identifying initial codes and developing a coding framework, (3) identifying potential themes, (4) matching themes to the supporting data, (5) defining and naming themes, and (6) extracting relevant themes and producing a description of findings [30,33]. SB and GW coded the data. A constructionist approach, focusing on social conditions (user profiles and context of use) and structural conditions (app features and delivery of content), was used to interpret the data.

## Results

### Overview

A total of 19 adults with T2D were interviewed (mean age 60 years, SD 14 years; 42% women). Additional participant characteristics are detailed in Table 1. Interview participants were older, more educated, had a lower baseline hemoglobin A_1c_, and used the app twice as much as those in the intervention arm of the MDC trial. The mean duration of the interviews was 51 min (range 29-79 min).

**Table 1:** Participants’ demographic and clinical characteristics and current app use.

Characteristics		MDC^a^ trial (intervention arm) sample (n=93)	MDC interview participants (n=19)
Gender (female), n (%)		44 (47)	8 (42)
Age (years), mean (SD)		55 (10)	60 (8)
**Education (highest level), n (%)**			
	Year 10	10 (11)	5 (26)
	Year 12 or apprentice	42 (45)	2 (11)
	Graduate/postgraduate	41 (44)	12 (63)
**Employment status, n (%)**			
	Paid employment	59 (64)	7 (37)
	Retired	22 (23)	11 (58)
	Unemployed or other	12 (13)	1 (5)
**Diabetes duration (years), n (%)**			
	<5	43 (46)	8 (42)
	>5 to 10	29 (31)	8 (42)
	>10 to 20	7 (8)	3 (16)
	Unknown	14 (15)	0 (0)
Hemoglobin A_1c_ (%), mean (SD)		7.3 (1.5)	6.8 (0.9)
Hemoglobin A_1c_ (mmol/mol), mean (SD)		56 (44)	51 (20)
**General app use, n (%)**			
	Multiple times per day	69 (74)	14 (74)
	Once a day	23 (25)	4 (21)
	Less than once a day	1 (1)	1 (5)
Interactions with the MDC app (number), mean (SD)		18 (15)	36 (17)

^a^MDC: My Diabetes Coach.

### Themes

A total of 2 high-level themes were constructed from the data: (1) the moderating effect of diabetes self-management styles on needs, preferences, and expectations and (2) factors influencing users’ engagement with the app: one size does not fit all. These comprised several subthemes, as described in the following sections (summarized in Textbox 1).

**Interview themes and subthemes.**

**Moderating effect of diabetes self-management styles on needs, preferences, and expectations**

- Self-directed versus externally directed self-management styles
- Group differences in app preferences

**Factors influencing users’ engagement with the app: one size does not fit all**

- Interaction mode preferences
- Minimizing disruption to everyday life
- Initiating engagement

### Theme 1: Moderating Effect of Diabetes Self-Management Styles on Needs, Preferences, and Expectations

This theme describes variations in self-management styles and how these influenced app preferences.

#### Self-Directed Versus Externally Directed Self-Management Styles

When asked to describe how they managed their diabetes and their diabetes knowledge before using the MDC app, participants expressed very different levels of autonomy, motivation, and efficacy. Of the 19 participants, 11 described themselves as having always had an independent, self-directed self-management style. For example, they were intrinsically motivated to seek diabetes-related information when they were first diagnosed, saying:

I'm a bit of a researcher because it's about my own health.

They also expressed confidence in their diabetes knowledge and self-care ability, describing themselves as experts in their own care and comparing themselves with “other people [with] diabetes [who] don't have as much knowledge.” A common shared characteristic was that they used their smartphones for “just about everything” and reported previously using health apps to help them achieve their health goals.

In contrast, the remaining 8 participants expressed a more externally directed style and did not engage in independent information seeking. Instead, they preferred to rely on their health professionals and diabetes organizations for diabetes-related information. They expressed less confidence in their diabetes knowledge, describing it as limited to “only what the doctor has told me.” As they did not seek diabetes information at diagnosis, they referred to being *“*very lost in the beginning, [because] nobody tells you anything.” Although most participants used computers and tablets, they were not as comfortable with smartphones, only using them for phone calls and text messaging: “the mobile, it's just for [an] emergency.” Consequently, these participants were less likely to report using other health apps.

When asked to describe their experiences with the MDC app, there were clear differences between participants expressing a self-directed versus externally directed self-management style in terms of their needs, preferences, and expectations.

#### Group Differences in App Preferences

The self-directed participants described how support via an app should ideally account for their existing diabetes expertise and be presented to enable them to have the final say in their care:

If I can summarize what I look for, it's not so much “tell me what the answers and solutions are, but give me the information, give me the options, I'm making this decision.” I'm not looking for hand holding.

Consequently, facilitating decision making by enabling easy tracking of multiple sources of health-related data was a key consideration. For example:

Track the things that I want to track, daily readings, weight, blood pressure, record medication [and] blood test results and probably 15 other things that are important to me. If you can't record something, you can't control something.

The purpose of tracking was to refine established routines and identify how specific actions, for example, taking certain supplements such as *Chromium Picolinate 400 mg*, related to *actual changes*, such as lowering blood glucose levels from *7.1 to 6.5.* The other purpose of tracking was to facilitate changes to self-management, for example:

When I'm making a change in my own practices: to closely monitor things when I'm increasing my exercise.

Curated, in-depth information was another vital feature for this group: “my motivation in using an app is [only] to get information*.*” They were interested in exploring a wide range of topics:

I'm interested in the technology of diabetes care, I'm interested in stuff all over the place, like reading about the impact of sugar on muscle.

It was important that the information was reliable, *like Cochrane Reviews* and *curated*, that is, organized in a way that enabled them to distinguish *basic information* from *in-depth discussion.*

Conversely, what was most helpful for participants with a more externally directed self-management style was not having to search out and evaluate diabetes information:

The information is provided, you don’t have to go searching for it, and that’s what’s convenient.

Without this easy access, one participant described how they “wouldn’t have looked [it] up... because lazy people don’t do that.” There were other instances where these participants described needing additional motivational support. For example, one person said they “get lazy,” and another said:

I'm one of these people - I go really good at something for a while, and then I get a bit slack and then I stop doing stuff.

This may explain why this group appreciated attempts at gamification and making learning fun, describing novel features of the app, such as IVR and the relational agent, as being “exciting,” “more interactive,” “cool and unique,” and increasing their *“interest.”* However, those who described a more autonomous self-management style were less receptive of attempts at increasing engagement such as gamification (eg, quizzes), which for them did not “add or detract from the experience” and were dismissed as examples of “the same information presented in a different way.”

Perhaps because of their experience using other health apps, the group expressing more self-directed self-management styles had higher expectations of the MDC app and were less tolerant of technical issues:

It has to be reliable because that's my expectation now of apps and other things and I can always find an alternative these days.

They expected flexibility in navigating through the MDC app in a way that suited them. For example, “a little less linearly,” with “a higher degree of user control in terms of being able to investigate down particular information paths and then back out of them.” They wanted the choice to be able to skip a particular topic if it was not “relevant” or “to go back over information” later through increased “searchability” if they found a topic particularly interesting.

On the other hand, participants from the other group did not have much experience with using apps and, therefore, were more forgiving of technical issues, for example, “just teething problems because it was so new.” However, because this group tended to limit their smartphone use to phone calls, they expected to be able to use the MDC app on their tablet device*:*

I'm one of these people that think a mobile phone is a mobile phone, and if I want to do anything else I go to the iPad.

### Theme 2: Factors Influencing Users’ Engagement With the App: One Size Does Not Fit All

This theme describes how participants engaged with the app, specifically the context, mode, frequency, and duration of interactions and the factors influencing these choices.

#### Interaction Mode Preferences: “I Could Read Quicker, So I Chose to Not Listen”

Participants could choose one of the multiple ways to interact with the MDC app. First, they could use the built-in IVR technology to listen to what the embodied conversational agent Laura said and respond using the microphone. Second, they could listen to what Laura said but respond by touching one of the options on the screen. Third, they could choose to ignore or mute Laura’s voice, read the text on the screen, and respond by touching an option on the screen.

The novelty of being able to interact with Laura using IVR was described by some as “exciting” and “more interactive.” However, most users, regardless of their self-management style, soon discontinued their use of IVR, choosing instead to read the text and respond by touching one of the options on the screen. The primary reasons were that IVR did not offer any obvious advantages and had some drawbacks. For example, using IVR as a mode of receiving and responding to messages within a session took much longer than reading the text and tapping in a reply:

There was nothing wrong with the pace of her speech, it was just that I could read quicker, so I chose to not listen to her.

Technical difficulties were also a hindrance:

She didn't understand me [laughs]. I found that frustrating.

The context of use also influenced the choices of users. For example, many described the IVR function as inconvenient because of their surroundings, for example, “I was always doing it in the bedroom in the morning when my husband was still in bed asleep” or “I didn't use it, because most of the time I was on the train.” Some participants also described talking to the phone as unnatural: “I think it just looked silly, to be talking to your phone.”

Giving the user a choice to opt out of using IVR and use other interaction modes was critical. As one participant put it:

If I had to have talked to her, I think I would have pulled out!

#### Minimizing Disruption to Everyday Life: “It Wasn't a Problem to Find a Half an Hour”

The MDC app required participants to complete a session with Laura once a week at a time that suited them. A weekly appointment suited most, as “any more would become a chore” or “just too much.” The discipline of a regular weekly appointment was viewed favorably because it increased commitment:

If I did it my own way, I wouldn't have done it. I think an appointment time kept me accountable.

Another positive attribute was that they mimicked offline appointments, encouraging automaticity:

It was like an appointment with a doctor or going out for dinner with friends. You knew that at 6:30 Friday, you had to sit down and talk to Laura.

Another participant said:

Even my grandchildren would say to me, oh grandma, it's Thursday, and you’ve got to speak to Laura. I structured things outside of those times because I knew that time was taken. I did things around that time because it was to me a standard appointment.

Those in paid employment appreciated being able to choose a time that suited them:

I'm glad I could choose a time that suited me.

They also valued the flexibility of being able to complete chats within a certain time frame:

[If I missed my time] that was easy to get around, because you had 24 hours to actually go in and have the chat with Laura.

On the other hand, those who were retired had a set time every week and made the chat part of their schedule, with little to no variation from 1 week to the next “I'm retired now [laughs], so what else do I do?” or “I'm a creature of habit, and I like things to be ordered and I like the regularity, [so] I put it in the calendar.”

For those with busy schedules, the fact that the MDC intervention was divided into 15 to 30 minute chats, over several months, was a benefit and compared favorably with face-to-face diabetes self-management education and support programs:

It wasn't a problem to find a half an hour. When you've got to go off to some of these diabetes [education things] it's four-and-a-half hours! You try and find four-and-a-half hours when you work a 16-hour day, it just doesn't work.

#### Initiating Engagement: “You Need to Get [the App] in Front of People When They're in the First Days”

Participants unanimously emphasized the importance of access to an app supporting self-management immediately after the diagnosis of T2D as a means to come to terms with their diagnosis:

You need to get that in front of people when they're in the first days, [and thinking] “Whoa, what just happened to me?!”

Participants suggested that having an “introduction to the basic stuff, in a fairly accessible manner,” resulted in “the greatest benefit” and “greatest impact and usefulness.”

Many participants described diabetes education as nonexistent or insufficient:

Other than being prescribed medication, there was really nothing to support

self- management

Others who had access described diabetes education as being “blunt, didactic stuff, do this, do that, do this,” with no attempt to account for their personal circumstances.

Insufficient time spent with the health care team was described as another barrier to receiving comprehensive information and understanding it:

I think for most people, they’re getting information [from the app] they wouldn’t otherwise have heard, unless their diabetes educators are very, very thorough, and you’re visiting them once a week, and we don’t do that. They [educators] don’t have the time for that. Your GPs don’t have the time to go through that information with you.

In some cases, the lack of education had the effect of delaying attempts at initiating lifestyle changes and self-management behaviors:

So, I was able to reject [my diabetes] and lived in a bit of denial. It took me quite a while to find and assemble a team of people that I felt could help me.

Participants consistently expressed the view that MDC would be “useful for someone who was newly diagnosed” to “help them transition”:

They need to be pointed in the right direction, because it will take them a while to find it if they're not pointed in that direction.

Many also acknowledged the potential role of health care professionals in facilitating access to and adoption of apps following diagnosis:

I would see a real benefit in ensuring that people like GPs, diabetic educators are made very aware of the app and that they actively engage patients on diagnosis with the app.

Another said:

The GP should be going, well here's your blood test results, download this app and learn what's happening and why it's happening.

## Discussion

### Principal Findings

This qualitative study investigated users’ experiences of a T2D self-management app accessed via their own smartphones over a 9-month period in the context of their everyday lives. We identified 2 main themes: (1) the moderating effect of diabetes self-management styles on needs, preferences, and expectations and (2) factors influencing users’ engagement with the app—one size does not fit all. We found that the needs, preferences, and expectations of diabetes self-management apps differed based on participants’ self-management styles. The broad implication is that, in addition to previously identified characteristics, such as age, gender, and socioeconomic status, self-management styles also influence engagement and need to be investigated further [16,34,35].

Participants expressing self-directed rather than externally directed self-management styles were more likely to be proactive in seeking diabetes-related information and using other health apps [36,37]. A possible explanation for this finding may be found in the literature on *health consciousness*, defined as the extent to which an individual takes ownership of their own health condition [38]. Our data are consistent with previous evidence suggesting that individuals who are more health conscious may also be more self-directed in their information-seeking behaviors and more proactive in managing their health [39].

Our findings corroborate previous research on the benefits of personalization and tailoring while providing preliminary evidence on *how* app preferences can be personalized based on a specific user characteristic—diabetes self-management style [21,26,36,40]. For example, participants expressing more self-directed styles value tools that assist them in making independent, informed decisions about their own care. This suggests that to engage these users, messaging within an app needs to be presented as volitional choices rather than explicit directives and needs to acknowledge the user as an expert in their own care [41]. Additional features that are likely to improve engagement by this group include in-depth, current, accurate information on a range of topics related to diabetes care; the ability to track, link, and interpret multiple sources of diabetes-related data; and a high level of flexibility in navigating the app.

Our study also corroborates previous research that shows that people who present a more externally directed self-management style may need additional encouragement to sustain engagement. Previous research suggests that changing attitudes to and motivations for diabetes self-management may be especially important for this group [3,42]. As diabetes is a self-managed condition, successful models of care, especially for those who are not intrinsically motivated, must focus on strategies that promote and maintain autonomy [43]. Strategies to improve engagement in this group could include gamification elements such as quizzes and features that promote accountability, such as goal setting and mechanisms that re-engage users, such as regular feedback [24,32,44,45]. It may also be useful to consider giving these users more customization choices, for example, the device they prefer, because many users were more comfortable with a desktop computer or tablet than with a smartphone.

Almost invariably, the participants did not use IVR because it did not provide any additional benefit. Our findings add to existing research that suggests that features, such as IVR, although novel and interesting initially, can deter or distract from the main objective of using an app over time, especially if they do not improve usability and require additional effort [46]. The implication is that novel features should be used with caution because they can be expensive to implement and may not have the expected benefit. At the very least, users need to choose to turn off features based on personal preferences. Optimizing functionality is key because ease of use and efficiency trump novelty when apps are used in the context of ongoing, real-world self-management of a chronic condition [27,44,47].

Many participants described receiving little to no diabetes education and support following diagnosis, and in some cases, this delayed engagement in self-management [48]. Making time for and having access to adequate face-to-face education and support are often challenging for people with newly diagnosed T2D [4,6,7]. Our data support previous research demonstrating that providing diabetes education and self-management support via an app could be a feasible and acceptable complement to clinical care [8-10,14]. Equally important is the suggestion that this support may be more successful in engaging people when accessed immediately following diagnosis [37,49,50].

Our findings suggest that the proposed contact frequency and duration (ie, weekly sessions of 15-30 min) was acceptable (even for those with busy schedules) and enhanced engagement, potentially through increasing accountability and automaticity [51]. Enabling users to choose a regular time fitting into their schedule and some flexibility in altering that time to fit with competing demands encouraged engagement. However, it was clear that some limits on how the app was used were considered beneficial, even necessary, as many described how complete freedom could result in disengagement. Appointment reminders were useful, but only to those with a busy schedule because those who described themselves as less busy (eg, retired) preferred set appointment times and considered them to be part of an established routine, for which they did not need a reminder.

Finally, our data suggest that although diabetes self-management apps may be helpful in initiating and maintaining self-management behaviors, people with T2D are more likely to engage with an app when it is endorsed by their health care professional. There is some evidence to suggest that although health care professionals think apps may be useful, sourcing evidence-based, high-quality apps from the thousands available on the app stores remains a challenge [50,52,53]. Thus, initiatives are needed to provide health care professionals with reliable resources that enable them to choose quickly from a curated selection of evidence-based diabetes self-management apps while matching them with the individual’s needs.

### Strengths and Limitations

A key strength of this study is that it was conducted in the context of a randomized controlled trial of the MDC app. In contrast with many previous trials of self-management apps, participants used the app *in the wild*, that is, in the context of their everyday lives via their own familiar devices, addressing some of the limitations of previous trials. Participants also had access to the app for up to 9 months, making it possible to explore their real-world use and changes over time. This was a significant strength relative to most previous research where participants only used an app once or for a short period (usually less than four weeks). The purposive interview sampling strategy was successful in recruiting participants with a range of experience, facilitating examination of the interplay between user characteristics, app preferences, and engagement. One exception is that expert app users and those expressing a more autonomous self-management style were overrepresented, perhaps because these characteristics made them more likely to want to participate in the interview study. In addition, interview participants used the app twice as much as those in the intervention arm of the MDC trial, suggesting that we were less successful at recruiting less engaged users. We recommend that future research focuses on identifying the experiences and needs of users who are less autonomous and less experienced with technology because they are likely to have different diabetes education and support needs. Finally, our sample did not include younger adults with T2D, a burgeoning cohort with clear unmet needs [54]. Further research is needed to explore the experiences of such a sample.

### Conclusions

Our study is one of the first to investigate the use of a diabetes self-management app in the wild. Our findings suggest several ways in which user experiences can be engineered to improve engagement with T2D self-management education and support via an app, such as personalizing app features to user characteristics, recommending a potential optimal time to intervene, developing resources to assist health professionals make evidence-based recommendations for diabetes apps, and recommend potential frequency and scheduling of the intervention. Further research investigating interactions between user characteristics, including self-management autonomy and engagement, is warranted to determine specific strategies to improve engagement with T2D self-management apps if diabetes self-management apps are to become a useful complement to clinical care.

## Appendix

Multimedia Appendix 1 COREQ Checklist.

## Abbreviations

IVR: interactive voice recognition
MDC: My Diabetes Coach
NDSS: National Diabetes Services Scheme
T2D: type 2 diabetes
